# Sustainability planning in the US response to the opioid crisis: An examination using expert and text mining approaches

**DOI:** 10.1371/journal.pone.0245920

**Published:** 2021-01-28

**Authors:** Carlos Gallo, Karen Abram, Nanette Hannah, Lauren Caton, Barbara Cimaglio, Mark McGovern, C. Hendricks Brown

**Affiliations:** 1 Department of Psychiatry and Behavioral Sciences, Feinberg School of Medicine, Northwestern University, Chicago, Illinois, United States of America; 2 Department of Psychiatry and Behavioral Sciences, Stanford University School of Medicine, Palo Alto, California, United States of America; 3 Illinois Department of Human Services, Division of Substance Use Prevention and Recovery, Chicago, Illinois, United States of America; 4 Department of Medicine, Stanford University School of Medicine, Palo Alto, California, United States of America; Universita degli Studi di Milano-Bicocca, ITALY

## Abstract

Between January 2016 and June 2020, the Substance Abuse and Mental Health Services Administration rapidly distributed $7.5 billion in response to the U.S. opioid crisis. These funds are designed to increase access to medications for addiction treatment, reduce unmet treatment need, reduce overdose death rates, and provide and sustain effective prevention, treatment and recovery activities. It is unclear whether or not the services developed using these funds will be sustained beyond the start-up period. Based on 34 (64%) State Opioid Response (SOR) applications, we assessed the states’ sustainability plans focusing on potential funding sources, policies, and quality monitoring. We found variable commitment to sustainability across response plans with less than half the states adequately describing sustainability plans. States with higher proportions of opioid prescribing, opioid misuse, and poverty had somewhat higher scores on sustainment. A text mining/machine learning approach automatically rated sustainability in SOR applications with an 82% accuracy compared to human ratings. Because life saving evidence-based programs and services may be lost, intentional commitment to sustainment beyond the bolus of start-up funding is essential.

## Introduction

The opioid crisis in the United States has continued to escalate. There were 46,802 total opioid related deaths in 2018, a historical high that continues to rise, with a provisional number of opioid related deaths exceeding 50,000 in 2019 [1]. An increasing number of individuals who die from opioids are also using stimulants such as methamphetamine and cocaine [2]. From 2015 to 2019, the death toll per 100,000 in the US population increased from 16.3 deaths to 595 deaths attributable to opioid overdose [3]. Despite some states’ recent reductions in opioid related mortality [4], there are signs that these rates will continue to increase at least in some areas, especially with the influx of synthetic opioids [5] and rising mixtures of opioids with methamphetamines and cocaine [2].

In December, 2016, the 21st Century Cures Act provided $1 billion in new funding to address the exponentially increasing opioid crisis [6]. Via the State Targeted Response (STR) grants, the Substance Abuse and Mental Health Services Administration (SAMHSA) began distributing these funds to the states through two allocations in April 2017 and March 2018. SAMHSA announced a third $1.5 billion funding opportunity called the State Opioid Response Grant (SOR) due in August 2018, and money was allocated in September 2019 to continue progress made by states and territories through the STR grants. For the purpose of this paper we refer to these as the 2018 SOR applications. The recently announced FY 2020 SOR budget has an equal amount of support [6]. The SOR grants aim to address the opioid crises by increasing access and infrastructure support for medication for treatment of opioid use disorder (MOUD, i.e., using the three FDA-approved medications for the treatment of opioid use disorder of methadone, buprenorphine, and naloxone); expanding services and reducing unmet prevention, treatment and recovery need; and reducing opioid overdose-related deaths from prescription opioids, heroin and synthetic opioids. SOR applications required a section dedicated to explaining sustainability plans. In particular, states were required to “Describe your plan to ensure these [Required] activities are sustained after grant funding ends” [7].

While the large influx of recent funding offers the potential for expanding states’ and territories’ ability to provide services, the question remains whether such expansions are likely to be sustained when the federal funding streams for this crisis start to decline. The goal of sustainability originally was defined as the extent to which an Evidence Based Intervention (EBI) can continue to deliver its intended benefit over an extended period of time after external support is finished [8, 9]. Recent empirical work on sustainability has led to an expansion of this definition. Thus sustainability can also refer to conditions that create lasting health outcomes by creating infrastructures during the time of funding support. For instance, Palinkas and colleagues wrote “the continued use of program components and activities for the continued achievement of desirable program and population outcomes… some definitions of sustainment focus on the continued existence of the coalition itself while others focus on the activities and impacts of the coalition” [8] after external support is finished. Sustainability was originally defined as the ability to maintain EBI components but has evolved to include a broader definition, including elements of ongoing collaborations/partnerships, infrastructure support, community need, ongoing evaluation of performance and outcomes, and availability of funding.

Recent research has contributed to conceptualizing and evaluating sustainability [10–15], but still lags far behind that of the other core phases of implementation science, namely exploration, preparation and implementation with fidelity [14]. This is not to say that sustainability planning is being ignored; sustainability planning was in fact a requirement of the State Opioid Response proposals. However, there is a general tendency during a period marked by financial expansion for governments to concentrate on initiating new implementation strategies at the expense of retaining these capacities beyond grant periods.

The focus of this paper is to measure how well states’ SOR grants included planning for sustainment, and whether state characteristics of population, severity of opioid use disorder, and availability of existing services influenced plans for sustainment. With a view towards the future in which automated procedures for classifying sustainability in similar documents could be used in place of labor-intensive human coding, we also used text mining and machine learning to examine the degree to which such automated procedures could replicate human coding. Similar procedures have been proposed [16] and developed [17] in implementation research to monitor implementation process, but this is the first time we know of that specifically addresses the use of these procedures for sustainability or for treatment of substance abuse.

## Methods

### Collection of State Opioid Response reports

This study was carried out through the support of a National Institute on Drug Abuse (NIDA) supplement to one of the authors (MM); it was classified as an exempt, non-human subject study by both the Northwestern University and Stanford IRBs. In seeking to determine the degree to which states have described their sustainability programs, we requested the first SOR applications from each of the 50 states. We chose to exclude territories and the District of Columbia from this study as their administrations were quite different from the states. Additionally, territories and the District of Columbia received a set, equal amount of support regardless of population characteristics or need. We used SAMHSA’s website [18] to find the email addresses of the single state agencies (SSAs) for substance abuse services in each state, and wrote directly to each state’s director [18].

We contacted SAMHSA to inform them of our plan to contact current state SSAs requesting full SOR applications and collect additional personnel contacts through former SSAs. We were guided by the National Association for State Alcohol and Drug Abuse Directors (NASADAD) in how to communicate these requests so as to minimize burden to busy state directors. All SSAs, as well as SAMHSA and NASADAD, were provided a summary of the research protocol. We informed all parties that all documents would be held in confidence, and the analyses would be reported in aggregate across states with no identification. All states’ provision of their SORs were completely voluntary. Neither SAMHSA nor NASADAD took an active part nor directly encouraged states to participate, but could answer questions if states contacted them. The funder, NIDA, also did not take an active part. A total of 34 states (68%) provided their SORs.

### Data abstraction from the SOR applications: Human coding of sustainability

For this paper we borrow from the recent empirical work of Palinkas and colleagues that identifies common elements of sustainment across multiple SAMHSA programs [8]. In this work, sustainability including elements of ongoing collaborations/partnerships, infrastructure support, community need, ongoing evaluation of performance and outcomes, and availability of funding. This work guided our selection of general categories organized into three major elements that we were able to code reliably from the SOR abstracts. Specifically, our category of “funding” was described as “availability of funding,” in that paper. Our category of “policies, partnerships, or regulations” included “collaborations/partnerships” and “infrastructure support” listed in that paper. Our category of “quality monitoring” mirrored “ongoing evaluation of performance and outcomes” in the recently published paper. We did not include the paper’s other category of future “community need” as some communities’ need was expected to remain high in every state, and projections of such need were absent from these SOR applications.

A team of five doctoral level coders were assembled to review the SOR applications using a standard protocol described below to assess the degree of sustainability across three critical categories: 1) *Funding*, 2) *Policies*, and 3) *Quality Monitoring*. These components were specified as follows:

1. **Funding**. Funding was considered sustainable if it included one or more of the following sources that was judged reasonable beyond the grant period: a) Medicaid 1115 waiver was described in the SOR, regardless of whether it is was approved or pending; b) State mentioned sources of funding from private insurance; c) The SOR described new CPT/Billing codes that allowed billing of insurance for opioid treatment services; d) SOR described a partnership with offices that already provide funded services for Drug Control Policy or Alliance of Recovery Residences; or e) existing funding streams supporting the Hub and Spoke model, which required high startup costs.
2. **Policies**. The SOR was considered to have sustainable policies when it presented new policies, partnerships or regulations of the state that supports the proposed program beyond the funding period. Ongoing partnership included different state agencies that were committed to continue providing services. These include criminal justice agencies, public health departments, and police departments. Other sustainable activities under this category include mandated trainings for all providers in opioid related services within private specialty clinics, federally qualified health centers (FQHC) and emergency rooms.
3. **Quality monitoring.** When quality adherence programs or ongoing program evaluation are explicitly referenced after funding ends, then the application is deemed to meet the quality monitoring criteria. This ongoing quality monitoring includes tracking patients on medication, tracking care coordination, linkage, retention, monitoring and feedback to ongoing learning collaboratives, and finally a description of a data-based evaluation system that has ongoing support.

As an example, a positive score of one (1) is given on *funding* when an SOR application met the criteria described above for funding, otherwise a score of zero (0) is given when it lacked an indication of sustainable funding or simply mentioned there would be future funding but gave no clear plans. We developed a new protocol for sustainability coding based on a full qualitative assessments of sustainability plans by interviewing previous and current single state authorities (SSAs) on recently published work [19]. The highest score of 5 was assigned to SOR proposals that met all three criteria for its clear discussions of sustainability. A 4 was assigned when only two sustainability criteria were met, and a 3 when only one criterion was met. If an SOR proposal had some discussion of sustainability but no definite plans were identified, a value of 2 was assigned. In other words, unless there was a clear indication of how a program would be sustained, that component of sustainability planning was scored as absent, with a value of 2. Finally, a value of 1 was given when there was no mention of sustainability at all. Three coders rated each SOR independently on this five-point scale. Once all SORs were individually scored, any disagreements were resolved by consensus [20]. Nearly all the initial individual scores agreed within 1 unit, and consensus was reached on all 34 reviews.

When an SOR application was assessed to have any sustainability discussion (a score of 2 to 5), then we coded which of five specific activities were being sustained. These five activities are: a) *Medications for Opioid Use Disorder* (MOUD) including Suboxone, Buprenorphine, Naltrexone, and Methadone. b) *Overdose Services*: Naloxone, syringe exchange programs. c) *Prevention Services* including harm reduction, screening for misuse and risk factors (e.g., SBIRT), primary prevention with drug abuse prevention programs, prescription drug monitoring program (PDMP), prescription disposal services such as drop-off and take back. d) *Maintenance of Recovery* involving behavioral interventions targeting social determinants of health, job skills, and physical health. e) *Implementation Support* includes strategies such as ECHO training, Hub and Spoke models, mentoring, coaching, learning collaboratives, and technical assistance.

### State measures tested as predictors of sustainability ratings

To investigate how states’ sustainability relates to general population characteristics, need for opioid services, and the service delivery capacity, each state was characterized along the following dimensions: state’s population characteristics, opioid metrics including prescription and mortality, and treatment service availability. Wherever possible, measures from these three dimensions were based on 2017 data, as the SOR applications were written and due in 2018, funded in 2019, but used, at the time, the most updated data, namely 2017’s data. Hence, 2017 information is used to predict sustainability ratings (Table 3), as well as to identify whether there was any sampling bias between the states that provided their SOR application and the states that did provide their SOR application (Table 1).

**Table 1:** Comparison of states characteristics depending on whether an SOR application.

	SOR States (N = 34)	No SOR States (N = 16)	
	Mean (StDev)	Mean (StDev)	p-value
**State Population Characteristics**			
Population (Log for pvalue)	6.9M (7.6M)	5.7M (6.7M)	0.585
Urban (%) in 2010	75.4% (14%)	69.7% (15.4%)	0.219
Minority (%)	31.2% (17.1%)	31.4% (13%)	0.962
Poverty (%)	12.9% (2.95%)	13.4% (2.85%)	0.550
Uninsured (%)	7.5% (2.62%)	9.5% (3.54%)	0.056
**Opioid Use, Misuse, Mortality**			
Opioid Prescriptions (100 people)	59.9 (14.1)	68.9 (21.8)	0.148
Misuse 12 and older (%)	4.55% (0.762%)	4.68% (0.72%)	0.551
Misuse 12 to 17 (%)	3.66% (1.14%)	4.49% (1.03%)	0.015 *
Misuse 18 to 25 (%)	8.21% (1.62%)	8.32% (1.85%)	0.840
Misuse 26 and older (%)	4.05% (0.86%)	4.07% (0.72%)	0.926
Admissions (100K people)	214 (229)	134 (127)	0.131
Opioid Deaths (100K people)	19.5 (10.7)	16 (9.03)	0.245
**Opioid Treatment Capacity and Utilization**			
SOR dollar per person	$4.29 ($3.79)	$3.36 ($2.61)	0.318
Methadone Maintenance Clinics (100K people)	0.61 (0.41)	0.50 (0.49)	0.433
Misuse per MM Centers (100K people)	8.12 (4.28)	11.7 (7.13)	0.082
Prescribing Physicians (100K people)	18.20 (7.93)	15.9 (9.84)	0.413
Medicaid Expansion Implemented in State	Yes = 27 (79.4%)	Yes = 9 (56.2%)	0.173
MOUD Service Covered in State	Yes = 27 (79.4%)	Yes = 13 (81.2%)	0.988

Source: Data was obtained from different sources CDC, Census Bureau, and SAMSHA.

Note1: State's descriptions are drawn from 2017 data when the SOR documents were created, except for Urban Percentage taken from 2010

**State Population Characteristics** were compiled from recent public records for 2017 [21]. The US Census datasets were used to examine state’s characteristics in population [22], and percentages of urban density percentage [22], minority population [23], people living in poverty [24] and uninsured individuals [25]. **State’s opioid use, misuse, and mortality rates** were collected in 2017 for number of opioid prescriptions per 100 persons, opioid misuse percentage for 12 years and older, along with specific age brackets of 12 years to 17 years, 18 years to 25 years, and 26 years and older [26], opioid hospital admissions per 100 thousand people [27], and opioid deaths per 100 thousand people [4, 28–30]. Other potentially useful variables such as synthetic opioid deaths [5, 31] were only available for a portion of states, leaving too much missing data for a careful analysis. Hence, such variables were excluded. To the extent possible, we verified data consistency. For instance, we treated a datum as missing when we could not resolve state’s reporting accuracy in numbers. Likewise, when hospital admissions were far greater than discharges rates we treated that state’s admissions values as missing (N = 1). **State’s treatment capacity and utilization** per 100 thousand people were measured by the SOR budget amount, number of methadone maintenance treatment centers per 100 thousand people [32–34], number of prescribing physicians [35], state Medicaid expansion status [36], and whether or not MOUD services were covered by insurance or Medicaid [28]. Additional variables such as copayment and limits on MOUD services [37] were not included in analysis due to significant missing data and high variation in eligibility for reduced rates.

MOUD services were collected from SAMHSA websites [38]. Data on the different types of drug types and deaths were collected from the CDC [29]. Other data specific to opioid and other prescription drugs were collected from KFF.org [33, 39]. Iowa Community Indicator Program was used to collect rural area data for the states [40]. Medicaid expansion and waiver information on the states was obtained from the National Conference of State Legislatures website and the KFF organization [41–43]. Medicaid Expansion is a provision in the Affordable Care Act (ACA) that calls for expansion of Medicaid eligibility to cover more low-income Americans [44]. Under the expansion, Medicaid eligibility would be extended to adults up to age 64 with incomes up to 138 percent of the federal poverty level. Each state determines whether to participate in the Medicaid expansion program. In keeping with our agreement not to identify states, we excluded variables where less than 5 states were coded in one category (e.g. 1115 Waiver Exception).

### Analytical procedures

We first examined (Table 1) whether those states providing their SOR applications differed on the full set of predictors mentioned above. Continuous data were compared using t-tests after suitable transformation (e.g., log for population); binary proportions were compared using χ2 tests, and count data (e.g., number of opioid treatment centers) were compared using Poisson regression, after adjusting for the relevant population (e.g., those with opioid use disorder). Because of the small number of states and corresponding modest power—a traditional 0.5 two-sided t-test has 70% power to detect a 0.8 effect size and similarly a χ2 test has this same power to detect a true difference of 0.2 and 0.55 –we also relied on diagnostic p-p plots to compare the observed p-values to those derived from a corresponding null distribution [45].

To examine how sustainability rating can be explained by state level predictors, we used ordinal regression (Table 3), which assumes that the log-odds based on all the splits into lower and higher scores on the 1–5 scale is the same (i.e., a cumulative logistic model) [46]. We report this log-odds estimate and its standard error; positive values indicate higher ratings of sustainability with higher values of the predictor variable. Tests of relationships with the sustainability score were based on univariate Wald-type tests of the log-odds estimate divided by its standard error, and we designate tests with p-values less than 0.05 as significant. In addition to the univariate tests, we conducted a backward stepwise multiple regression model that incorporated all predictor variables that reached 0.20 significance, and dropping variables that had substantial multicollinearity. In analyzing binary outcomes (e.g., whether MOUD was sustainable), we used standard logistic regression.

### Assessing sustainability using automatic methods: Text mining and machine learning

#### Text mining

As the human coding of sustainability requires substantial effort, we explored whether accurate automated classifications of sustainability could be obtained (Table 4). Recognizing that 34 documents is a small number to use text mining to classify states into 5 categories to extract key words and phrases, we chose to dichotomize the sustainability scale into two categories: 4–5 versus 1–3. Text mining searches identified specific linguistic content pertaining to words, chosen by the authors and implementation and opioid experts, in the following categories: education, prevention, rescue, interventions, implementation and sustainability. For instance, the sustainability category contained the terms: sustaining, sustainment, sustainability, and beyond funding, among others (see S1 Appendix-Content for lists of content-related words). The implementation category contained: acceptability, adoption, feasibility, fidelity, among others. Evaluation terms included: monitor, assessment, and feedback. We included 8 categories of implementation strategies from a recent classification system [47]. We also tested to see whether automatically extracted linguistic features related to *content* and *style* can predict sustainability as assessed by human raters. The list of keywords used in these categories is located in S1 Appendix-Content. Linguistic features in both content and style have been used to predict success of funding in crowdsourcing campaigns [48] and academic success [49]. We tested two linguistic dimensions as predictors of sustainability using multiple regression modeling: 1) function words (articles, prepositions, auxiliary verbs), and 2) verb tenses in future form. To control for document length, each count of linguistic features was divided by the text length. These stylistic indicators were examined the machine learning algorithm described below.

#### Machine learning

Machine classifier algorithms were also tested to explore if sustainability could be predicted in an automatic fashion. Support vector machines (SVM) [50] were trained to identify sustainability ratings based on the language patterns found in the SOR application documents. The SVMs were trained and tested using cross validation with 1-hold-out [51] where one SOR application selected at random was used for testing the algorithm prediction. The rest of the SOR applications were used as training data. The accuracy results were averaged over the cross-validation samples. This setup aimed to make most of the small sample size while reducing potential bias from training and testing document selection. The SVM algorithm has several parameters that can be determined empirically based on the nature of the dataset. The parameters explored in this paper are the kernel used to transform the data into a separable hyperplane (radial, linear, and linear with cost choice), and the cost of misclassification (C). The kernel function transforms the input data into a different space so that classification can be accomplished by a hyperplane [52]. The optimal hyperplane separates the training data using a maximum margin. We can allow misclassifications by controlling the size of the margin. A large margin allows more misclassifications. A narrow margin allows fewer misclassifications. This is called the “cost” function. The optimal cost of misclassification can be found by comparing classification performance and trial and error of the cost value [53].

SVM classifiers do not provide interpretable coefficients; however, they are useful in recognizing categories in unseen data. We also tested additional algorithms such as the Multinomial Naive Bayes classifier implemented in the Weka machine learning toolkit [54]. The independent variables included a more comprehensive set of indicators for the structure and form of the language, including words related to articles, propositions, words longer than 6 letters, number of words per sentence, verb tenses to measure a focus on past, present or future, propositions, articles, and auxiliary verbs.

## Results

**Fig 1:**
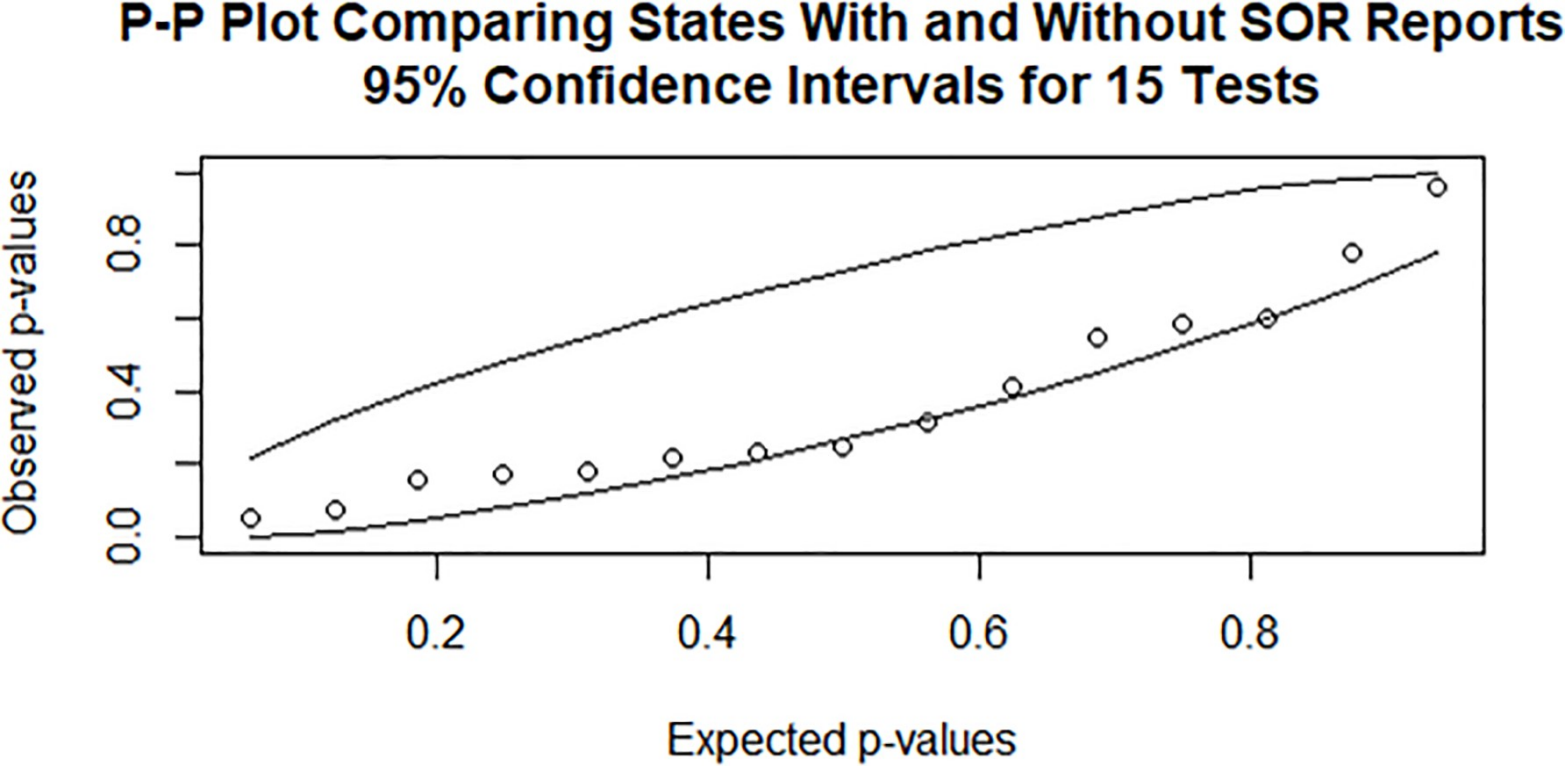
P-P plot comparing states with and without SOR reports.

Table 1 compares two groups: 1) states with SOR documents (N = 34) and 2) states without an SOR document available (N = 16). The comparison was done using a t-test and chi-square significant tests across 15 characteristics of the state’s population, opioid use and mortality, or treatment capacity. Overall we found very limited differences between the two groups (i.e. states with SORs applications versus states without available SORs applications). The only dimension for which a significant difference was found was the percentage of opioid misuse among youth ages 12 to 17 years old (p = 0.015), with 3.66% of states with available SORs versus 4.49% for those without. This significant comparison is not surprising given the 15 multiple tests conducted at the 0.05 level. We examined this further with a p-p plot (Fig 1), which compares the observed ordered p-values against what one would obtain by simulation under a null distribution of 15 independent uniform random variables. In this plot, the observed 15 p-values all fell within or adjacent to the 95% confidence interval from simulation, and there was no indication that there were smaller than expected p-values at the left end of the plot. Thus we found minimal indication of bias selection in the available set of 34 states with SORs documents.

Table 2 describes the sustainability distribution of the 34 SOR applications that were available. The scale used for rating sustainability ranged from a lowest of 1 to a highest of 5 with the following frequencies: Grade 1 was assigned to three states (8.8%), grade 2 to eight states (23.5%), grade 3 to eleven states (32.3%), grade 4 to ten states (29.4%), and the highest grade 5 to two states (5.9%). Thus one-third had a rating of 4 or 5, and a similar proportion provided no evidence that any of the three criteria had plans to be sustained. The three criteria necessary for sustainability had the following distribution. Funding criterion was described by 20 states (58.8%), Policies criterion by 16 states (47.1%), and Quality Monitoring criterion by 3 states (8.8%). The main five activities sustained in the SORs were services related to MOUD service activities in 25 states (73.5%), Overdose service activities in 16 states (47.1%), Prevention service activities in 23 states (67.6%), Maintenance service activities in 23 states (67.6%), and Implementation support service activities in 16 states (47.1%).

**Table 2:** Distribution of sustainability ratings in 34 SOR documents.

Sustainability Scale (N = 34)	Three criteria met	Two criteria met	One criterion met	Sustainability Mentioned without details	Sustainability Not mentioned
	(5 grade)	(4 grade)	(3 grade)	(2 grade)	(1 grade)
	N = 2 (5.9%)	N = 10 (29.4%)	N = 11 (32.4%)	N = 8 (23.5%)	N = 3 (8.8%)
**Sustainability Criteria**	**Funding**	**Policies**	**Quality Monitoring**		
	**N = 20 (58.8%)**	**N = 16 (47.1%)**	**N = 3 (8.8%)**		
**Sustainability Activity**	**MOUD**	**Overdose**	**Prevention**	**Maintenance**	**Implementation Support**
	**N = 25 (73.5%)**	**N = 16 (47.1%)**	**N = 23 (67.6%)**	**N = 23 (67.6%)**	**N = 16 (47.1%)**

Source: Data collected by authors

Table 3 summarizes the coefficient and standard error (SE) estimated by univariate prediction of the dependent variables; specifically the sustainability scores, criteria and activities. Significant predictors are shown in the rows with stars. The predictor variables are the state population characteristics, opioid use, misuse and mortality, and the opioid treatment capacity and utilization. The *Sustainability rating* was significantly likely to be higher when there was a larger number of opioid prescriptions per 100 people (Coef = 0.052, SE = 0.024, p = 0.03), higher percentage of opioid misuse in people 12 years and older (Coef = 1.281, SE = 0.476, p = 0.007), and higher percentage of the opioid misuse in people ages 18 to 25 years old (Coef = 0.845, SE = 0.261, p = 0.001). The *funding criteria* was more likely to be described in the SOR when there was a higher percentage of poverty in the states (Coef = 0.364, SE = 0.176, p = 0.034), higher percentage of opioid misuse in people 12 years and older (Coef = 1.463, SE = 0.621, p = 0.018), as well as those ages 26 years and older (Coef = 1.158, SE = 0.559, p = 0.038). Both the *policies* and *quality monitoring* criteria were more likely to be described in the SOR when there was a higher percentage of opioid misuse among those ages 18 years and 25 years old (Coef = 0.631, SE = 0.277, p = 0.023) and (Coef = 1.225, SE = 0.586, p = 0.037), respectively.

**Table 3:** Predicting sustainability, three criteria for sustainability, and five activities based on states characteristics, opioid use, misuse, mortality, and opioid service capacity and utilization.

		Outcome (Dependent Variable)								
		SUSTAINABILITY	THREE DIMENSIONS OF SUSTAINABILITY			FIVE ACTIVITIES BEING SUSTAINED				
			Funding Criteria	Policies Criteria	Quality Monitoring Criteria	MOUD Activity	Overdose Activity	Prevention Activity	Maintenance of Recovery Activity	Implementation Support Activity
List of Predictors		Coef (SE) ***	Coef (SE) ***	Coef (SE) ***	Coef (SE) ***	Coef (SE) ***	Coef (SE) ***	Coef (SE) ***	Coef (SE) ***	Coef (SE) ***
**State Population Characteristics**										
	Population (Log)	0.184 (0.287)	0.274 (0.342)	-0.138 (0.332)	-0.108 (0.58)	-0.122 (0.377)	-0.535 (0.359)	-0.424 (0.376)	-0.221 (0.337)	0.119 (0.332)
	Urban (%)	-0.005 (0.021)	-0.01 (0.026)	-0.012 (0.025)	0 (0.044)	-0.030 (0.031)	-0.024 (0.026)	-0.012 (0.027)	-0.01 (0.025)	0.025 (0.026)
	Minority (%)	0.005 (0.018)	0.008 (0.021)	-0.001 (0.020)	-0.047 (0.049)	-0.009 (0.023)	-0.004 (0.020)	0.023 (0.024)	-0.005 (0.021)	0.006 (0.02)
	Poverty (%)	0.204 (0.108)	0.364 (0.172) *	0.121 (0.123)	-0.053 (0.219)	0.492 (0.226) *	0.292 (0.148) *	0.107 (0.135)	0.029 (0.119)	0.031 (0.118)
	Uninsured (%)	0.072 (0.125)	0.176 (0.148)	0.071 (0.134)	0.027 (0.23)	0.658 (0.285) *	0.377 (0.171) *	0.215 (0.164)	0.099 (0.136)	0.272 (0.154)
**Opioid Use, Misuse, Mortality**										
	Opioid Prescriptions (100 people)	0.052 (0.024) *	0.054 (0.029)	0.042 (0.027)	0.032 (0.044)	0.083 (0.039) *	0.074 (0.032) *	0.052 (0.031)	0.035 (0.026)	0.041 (0.027)
	Misuse 12 and older (%)	1.281 (0.476) **	1.463 (0.621) *	0.722 (0.502)	0.597 (0.784)	1.496 (0.695) *	1.081 (0.552) *	2.061 (0.766) **	1.104 (0.558) *	1.700 (0.664) **
	Misuse 12 to 17 (%)	0.417 (0.293)	0.471 (0.341)	0.385 (0.328)	0.355 (0.545)	0.452 (0.373)	0.281 (0.318)	0.354 (0.344)	-0.069 (0.309)	0.312 (0.321)
	Misuse 18 to 25 (%)	0.845 (0.261) **	0.457 (0.258)	0.631 (0.277) *	1.225 (0.586) *	0.736 (0.351) *	0.235 (0.223)	2.089 (0.809) **	0.277 (0.227)	0.161 (0.219)
	Misuse 26 and older (%)	0.765 (0.396)	1.158 (0.559) *	0.293 (0.416)	-0.074 (0.729)	0.949 (0.587)	0.876 (0.493)	1.110 (0.581)	0.989 (0.513)	1.586 (0.631) *
	Admissions (100K people)	-0.001 (0.002)	-0.001 (0.002)	-0.001 (0.002)	-0.014 (0.016)	-0.001 (0.002)	0.001 (0.002)	-0.002 (0.002)	0.001 (0.002)	0.000 (0.002)
	Opioid Deaths (100K people)	0.013 (0.029)	0.028 (0.035)	-0.011 (0.033)	-0.052 (0.072)	-0.022 (0.036)	-0.018 (0.033)	-0.070 (0.039)	0.043 (0.035)	-0.029 (0.034)
**Opioid Treatment Capacity and Utilization**										
	SOR Dollars per person	-0.039 (0.074)	0.045 (0.099)	-0.111 (0.109)	-1.196 (1.143)	0.089 (0.133)	0.035 (0.093)	0.005 (0.099)	0.197 (0.128)	0.022 (0.092)
	Methadone Maintenance Clinics (100K people)	-0.397 (0.733)	-0.640 (0.851)	-0.326 (0.846)	-3.779 (3.128)	-0.233 (0.919)	0.736 (0.865)	0.234 (0.914)	1.931 (1.053)	-0.330 (0.847)
	Misuse per MM Centers (100K people)	0.098 (0.075)	0.078 (0.088)	0.072 (0.085)	0.270 (0.158)	0.077 (0.100)	-0.007 (0.083)	0.092 (0.095)	-0.091 (0.088)	0.064 (0.084)
	Prescribing Physicians (100K people)	0.029 (0.041)	0.052 (0.048)	0.014 (0.044)	-0.126 (0.113)	0.018 (0.051)	0.050 (0.046)	-0.008 (0.047)	0.098 (0.051)	-0.026 (0.045)
	Medicaid Expansion Implemented in State	0.570 (0.769)	0.087 (0.858)	0.214 (0.855)	NA (Note1)	-0.927 (1.159)	-1.291 (0.924)	0.577 (0.872)	-0.662 (0.858)	-0.511 (0.856)
	MOUD Service Covered in State	-0.432 (0.752)	-0.693 (0.922)	-1.291 (0.924)	-0.734 (1.306)	-0.927 (1.159)	-0.511 (0.856)	-1.261 (1.151)	1.866 (1.147)	0.214 (0.855)

Source: CDC, SAMSHA, Census Bureau

Note2: Likelyhood ratio test was used because of zero cell values, p = 0.227

With respect to the five activities that were sustained, we found the following: *MOUD service activities* were more likely to be described in the SOR application when there was a higher percentage of opioid misuse by those ages 18 to 28 years (Coef = 0.736, SE = 0.351, p = 0.036). *MOUD service activities* and *Overdose service activities* were both more likely to be present when the following four (4) predictors were higher, namely: 1) with higher percentages of populations under the poverty line (Coef = 0.492, SE = 0.226, p = 0.037) and (Coef = 0.292, SE = 0.148, p = 0.049), for MOUD and Overdose outcomes respectively, 2) with higher percentages of uninsured populations (Coef = 0.658, SE = 0.285, p = 0.021) and (Coef = 0.377, SE = 0.171, p = 0.028), for MOUD and Overdose outcomes respectively, 3) with higher opioid prescriptions per 100 people (Coef = 0.083, SE = 0.039, p = 0.03) and (Coef = 0.074, SE = 0.032, p = 0.019), for MOUD and Overdose outcomes respectively, and, finally, 4) with higher percentages of opioid misuse among those ages 12 years and older (Coef = 1.496, SE = 0.695, p = 0.031) and (Coef = 1.081, SE = 0.552, p = 0.05), for MOUD and Overdose outcomes respectively. *Prevention service activities* were more likely to be described when there was a higher percentage of opioid misuse among those ages 12 years and older (Coef = 2.061, SE = 0.766, p = 0.007), and from those ages 18 to 25 years old (Coef = 2.089, SE = 0.809, p = 0.01). *Maintenance of recovery service activities* were more likely among those with a higher percentage of opioid misuse among those ages 12 years and older (Coef = 1.104, SE = 0.558, p = 0.048). *Implementation support activities* were also more likely among states with a higher percentage of opioid misuse among those ages 12 years and older (Coef = 1.7, SE = 0.664, p = 0.01), as well as those with ages 26 years and older (Coef = 1.586, SE = 0.631, p = 0.012).

To extend the univariate tests presented in Table 3, we conducted stepwise multiple regression models that began with all predictor variables that reached significance of up to 0.20 (p< = 0.2), then removed if non-significant when included with this set of predictor variables. Predictors that were colinear above .9 were removed from the multiple regression model. Only one such case occurred, namely, opioid misuse among ages 12 and older and misuse among ages 26 and older had a correlation of 0.94. We kept the former variable, and removed the latter. In the multiple regression model treating *Sustainability Ratings* as the ordinal dependent variable, two predictors remained. Sustainability score was more likely to be higher when state’s population is poorer (Coef = 0.444, SE = 0.18, p<0.014), and with a higher percentage of opioid misuse among those 12 years and older (Coef = 0.695, SE = 0.305, p = 0.023). Of the three criteria of sustainability, again we removed the opioid misuse among ages 26 and older due to collinearity with those ages 12 and older. *Funding criteria* was more likely to be described in states with higher percentage of population living under poverty line (Coef = 0.809, SE = 0.381, p = 0.034). No other variables reached 0.05 significance regarding funding. In the multiple regression for Policies criteria and Quality Monitoring criteria, no variable reached significance. Of the five activities being sustained, Overdose activities were more likely to be described in states with smaller populations (Coef = -1.93, SE = 0.86, p = 0.025), and with a higher percentage of opioid misuse among those 12 years and older (Coef = 3.436, SE = 1.488, p = 0.021). Here again we removed collinear predictor opioid misuse among 26 years and older. No other variables reached significance in predicting Overdose activity. Namely, *Implementation Support activities* were more likely among states with higher percentage of uninsured population (Coef = 0.477, SE = 0.218, p = 0.036), and with higher percentage of opioid misuse among those 12 years and older (Coef = 2.477, SE = 1.006, p<0.014). No other variables reached significance.

To examine sustainability related activities more closely, we also ran a multivariable regression with the five activities as predictors of Sustainability score. While significance effects were not found, the univariate models showed that MOUD, Prevention, Implementation Sustainment Support activities were most closely related to Sustainability Score.

We now turn to the machine learning models. Table 4 summarizes the coefficient and standard error estimated by predicting the dependent variables of sustainability scores, criteria and activities using key words pertinent to broad classes of implementation strategies. In particular, the independent variables are counts of in-house selected words related to education, prevention, rescue, intervention, implementation, sustainability. In addition, we borrowed words in 8 categories from a recent classification system [47], and two linguistic features related to style and future verb tense. *Sustainability ratings* were more likely to be higher with lower word counts of Category 1 Evaluation (Coef = -4.037, SE = 1.506, p = 0.007). *Funding criteria* was more likely to be described in the SOR application when there were higher word counts in Category 7 Financial Strategies (Coef = 12.676, SE = 6.396, p = 0.047), and when there were lower words counts in Prevention (Coef = -15.17, SE = 6.777, p = 0.025), Category 1 Evaluation (Coef = -6.076, SE = 2.339, p = 0.009), and Category 6 Consumer Engagement (Coef = -11.273, SE = 5.16, p = 0.029).

**Table 4:** Predicting sustainability, three criteria for sustainability, and five activities based on linguistic features extracted from 34 SOR documents.

			Outcome (Dependent Variable)								
			SUSTAINABILITY	THREE DIMENSIONS OF SUSTAINABILITY			FIVE ACTIVITIES BEING SUSTAINED				
				Funding Criteria	Policies Criteria	Quality Monitoring Criteria	MOUD Activity	Overdose Activity	Prevention Activity	Maintenance of Recovery Activity	Implementation Support Activity
List of Predictors	Mean Words	Std Dev Words	Coef (SE) ***	Coef (SE) ***	Coef (SE) ***	Coef (SE) ***	Coef (SE) ***	Coef (SE) ***	Coef (SE) ***	Coef (SE) ***	Coef (SE) ***
**Education**	5.15	4.05	-2.673 (3.866)	-5.749 (4.786)	1.022 (4.497)	5.891 (6.776)	-2.230 (4.911)	1.022 (4.497)	-1.099 (4.728)	2.163 (4.529)	0.819 (4.494)
**Prevention**	4.56	3.53	-8.320 (5.010)	-15.170 (6.777) *	-4.166 (5.540)	-15.616 (16.162)	4.341 (6.631)	3.113 (5.402)	0.183 (5.722)	3.808 (5.425)	-7.066 (5.839)
**Rescue**	1.62	1.72	-7.994 (12.033)	-7.978 (13.646)	-13.654 (14.180)	6.410 (22.470)	-21.266 (14.995)	-9.725 (13.868)	-29.224 (15.287)	-19.896 (15.121)	-15.692 (14.376)
**Intervention**	77.09	28.56	0.557 (0.696)	0.348 (0.792)	0.480 (0.783)	-0.752 (1.426)	1.579 (1.070)	1.543 (0.908)	-0.051 (0.820)	0.065 (0.774)	-0.124 (0.771)
**Implementation**	5.5	3.39	-7.793 (5.540)	-6.987 (6.634)	-11.100 (7.340)	-2.344 (11.595)	-2.750 (6.993)	-1.219 (6.327)	-10.848 (7.155)	-10.869 (7.407)	-5.356 (6.585)
**Sustainability**	8.62	4.17	5.136 (4.858)	6.817 (5.377)	3.302 (5.161)	2.092 (9.158)	8.837 (6.015)	3.569 (5.173)	-2.501 (5.516)	2.312 (5.157)	10.328 (5.777)
**Category 1 Evaluation**	35.82	13.81	-4.037 (1.506) **	-6.076 (2.339) **	-1.976 (1.548)	-2.199 (2.575)	-2.359 (1.764)	-0.945 (1.457)	-1.932 (1.631)	-2.669 (1.651)	-3.872 (1.857) *
**Category 2 Assistance**	5.26	3.99	4.763 (4.073)	8.085 (6.002)	5.614 (5.125)	0.100 (8.228)	11.803 (8.097)	-4.152 (5.036)	-2.002 (4.892)	-1.646 (4.815)	10.945 (6.189)
**Category 3 Adaptation**	0.35	0.81	-41.695 (32.430)	-3.195 (36.762)	-1.947 (2.294)	NA (Note2)	-4.882 (40.294)	49.48 (42.054)	-21.007 (37.193)	-2.375 (36.752)	18.882 (37.059)
**Category 4 Partnership**	8.5	4.76	-3.728 (3.631)	-2.498 (3.895)	-3.626 (3.981)	-4.098 (7.548)	-3.932 (4.278)	1.151 (3.828)	-3.579 (4.077)	-3.626 (4.023)	-3.945 (4.008)
**Category 5 Education**	24.71	13.37	-0.213 (1.294)	-1.739 (1.432)	-0.302 (1.364)	5.079 (2.579) *	-0.729 (1.504)	-1.488 (1.440)	-2.270 (1.502)	-0.311 (1.374)	-0.716 (1.381)
**Category 6 Consumer Engagement**	8.35	4.63	-4.647 (3.972)	-11.273 (5.16) *	-0.339 (4.246)	1.981 (7.291)	-2.487 (4.755)	3.673 (4.340)	-1.771 (4.503)	0.770 (4.263)	-6.464 (4.607)
**Category 7 Financial Strategies**	6.44	4.19	7.519 (4.489)	12.676 (6.396) *	3.270 (5.048)	0.248 (8.743)	11.555 (7.153)	0.015 (4.986)	2.353 (5.442)	0.406 (5.009)	5.892 (5.212)
**Category 8 Infrastructure**	3.32	2.08	-4.568 (8.404)	-7.478 (9.838)	1.183 (9.564)	0.333 (16.818)	7.934 (11.106)	2.099 (9.572)	-8.149 (10.358)	1.467 (9.615)	-9.026 (9.805)
**Linguistic style (articles, propositions, etc)**	1678.71	401.14	-0.053 (0.055)	-0.045 (0.079)	-0.108 (0.103)	0.224 (0.249)	0.078 (0.074)	0.048 (0.076)	-0.011 (0.072)	-0.191 (0.137)	0.057 (0.080)
**Verbs in future tense**	97.74	31.34	0.152 (0.687)	0.536 (0.778)	-0.088 (0.746)	-0.949 (1.112)	1.880 (1.090)	0.352 (0.766)	1.042 (0.866)	-1.399 (0.955)	2.111 (1.164)

Source: Linguistic features generated by authors

Note3: Likelyhood ratio test was used because of zero cell values, p = 0.268

*No variable reached significance when predicting Policy criteria*. *Quality Monitoring* criteria was more likely to be present with higher number of words describing Category 5 Education (Coef = 5.079, SE = 2.579, p = 0.049). *Implementation Support* was more likely to be described in the SORs that had fewer words pertinent to Category 1 Evaluation (Coef = -3.872, SE = 1.857, p = 0.037). We found no significant predictors for *Policies*, *MOUD*, *Overdose*, *Prevention*, and *Maintenance of recovery activities*.

In addition to the univariate regressions, we conducted multiple regression models with all predictor variables that with significance below p< = 0.20 in the univariate model in Table 4. The correlation among these variables was under 0.4, and hence no variable with p value less than 0.2 was removed from the analysis due to collinearity. Sustainability was modestly related to Evaluation Category 1 words. Sustainability was higher with lower number of words within Evaluation Category 1 (Coef = -3.536, SE = 1.836, p = 0.054). For the five activities to be sustained, Implementation support activity was significantly more likely to be described when there were less words in Evaluation Category 1 (Coef = -5.595, SE = 2.657, p = 0.035). Prevention services activity was modestly related to the number of words describing Rescue activities. Prevention was less likely to appear when more words of Rescue activities were mentioned (Coef = -32.885, SE = 17.148, p = 0.0551). We found no significant predictors for *Funding*, *Policies*, *and Quality Monitoring*, *MOUD*, *Overdose*, and *Maintenance of recovery activities*.

### Using machine learning to predict sustainability

Three Support Vector Machines (SVM) implemented in R with kernels radial, linear, and linear with cost choice achieved an accuracy of 17%, 52%, and 78% respectively. We tested the optimal cost choice number from 0 to 1, and found that .63 leads to the lowest number of misclassifications. This cost choice leads to an accuracy of 78%. Additionally, a Naive Bayes Multinomial implemented in WEKA, reached an 82% accuracy in predicting Sustainability ratings.

## Discussion

Grant applications were requested from all 50 states in order to assess their sustainability commitments beyond federal funding; 34 states shared their application documents. There was no measurable bias between SOR and non-SOR states (Table 1). A protocol for assessing commitment to sustainability was developed to understand state’s response to the opioid crisis in their respective populations. We identified three criteria for sustainability beyond the funding period: identification of funding sources, development of policies, and assurance of quality monitoring.

Despite being a requirement of the SOR application, most states reported limited details on sustainability. Specifically, one third had no clear or no description of how they would sustain their programs. Another third met at least two of the criteria for sustainability, and only 2 states (5.9%) met all 3 criteria (Table 2).

A statement requiring a sustainability section in an SOR application is insufficient for understanding how states plan to sustain their strategies. While new research instruments are just beginning to examine sustainment [55], this area has been under-researched and under-practiced. This suggests that leaders are not fully equipped to enact policies and practices that will promote uninterrupted services and are unlikely to be coordinated with statewide efforts to scale up. Additionally, a state application that said state leaders intend to implement a policy does not guarantee that the policy will in fact be implemented. Our coding system was more rigorous than simply saying that a state intended to sustain programs. Unless there was a clear indication of how a program would be sustained, that component of sustainability was scored as absent. A full qualitative assessments of sustainability plans by interviewing previous and current single state authorities (SSAs) has also analyzed the context, intentions and difficulties on implementing sustainability plans on a recently published paper [19].

States with higher sustainability ratings generally had a higher proportion of the population receiving opioid prescriptions, as well as higher proportions with misuse particularly among young adults. Regarding the three sustainability criteria, funding was more likely to be described in the SOR applications for states having higher poverty rates and opioid misuse, especially among those 26 and older. Policies as well as quality monitoring were more likely to be present among states with higher rates of opioid misuse among young adults. Regarding the five activities, MOUD services were predicted by higher rates of poverty, uninsured citizens, opioid prescriptions and opioid misuse. Prevention services were more likely to be described in states that had higher misuse among youth and young adults. Maintenance of recovery and implementation support were more likely described when opioid misuse was high. In sum, states with the most opioid prescriptions, opioid misuse, and poverty generally had higher sustainability ratings, criteria, and activities.

Text mining can be used to extract linguistic features automatically without human actions, thus providing an opportunity to classify SOR and related documents rapidly and cheaply. Findings from standard text mining showed minimal or sometimes counter-intuitive directional relationships with sustainability measures. This may be due to the inability for single words have to reflect complex concepts such as sustainability. However, results from more complex machine learning algorithms were remarkably successful in predicting the sustainability ratings obtained by expert human raters. We found that 82% of the ratings were accurately predicted by the machine learning method, which provides a more sophisticated decision rule than that using individual word counts by themselves. This represents a useful first step towards measuring sustainability using automatic methods which can facilitate rapid assessments, and hence overcome common bottlenecks from using expert human raters. Other machine learning applications include the recognition of applied prevention research in the NIH portfolio [56]. In this work, Villani et al (2018) developed two machine learning methods with the goal of recognizing prevention studies in the NIH portfolio. Their accuracy ranged from 81% to 88%. Thus our performance of 82% is comparable. The NIH portfolio of prevention studies reached a sample size of N = 3814 which is several order of magnitude bigger than our data set. Machine learning algorithms do learn and improve as more data is available to train and test on.

It is important to interpret these findings through the lens of the state agencies responsible for these time-limited grants and ultimately the addiction prevention and treatment services in their jurisdiction. With the 21^st^ Century Cures Act bolus of funding, states were under pressure to spend funds rapidly. Thus, systems leaders may not have had adequate time or opportunity to plan beyond start-up. The mechanisms to achieve lasting policy and financing changes may be elusive to state leaders who often encounter changing policies at executive and legislative levels of government. These state leaders are situated in varying levels of government, from departments to divisions to bureaus that often have limited authority or capacity to communicate and align long-term strategies. We suggest that state authorities may benefit from sustainability strategies that establish and maintain inter-governmental alliances (e.g., leadership and data sharing across substance abuse care, mental health care, indigent care, child welfare, criminal justice, and emergency medical services). Factors of poverty, opioid treatment deserts, stigma, and discrimination all have pervasive influences on substance abuse and its treatment, so broader systems and policies related to housing, transportation, and education need to be coordinated in a system of systems approach. Drivers of sustainable policies and financing of evidence-based treatments are likely to require sophistication in politics, communication platforms, and continued engaging with advocacy, faith, and other community groups.

To date, implementation research has primarily accumulated knowledge on how to overcome barriers at the organizational or individual health care provider-level within specific macro level contexts [57]. In this paper we did examine widely different contexts based on population and service characteristics. However, it is also likely that higher state-level organizational structure can influence sustainment of opioid treatment and prevention programs. Further, the functioning of governmental as well as service delivery systems can change dramatically under stress, such as economic downturns and the recent COVID-19 pandemic. Thus an important limitation of this paper is that potentially relevant governmental structure and functioning dimensions are not included. This is because we were not able to locate accurate administrative or other data to reflect states’ recent organizational structures and functioning similar to that used to predict local government information sharing [58]. For future research in discriminating successful versus unsuccessful sustainment, it would be useful to include measures of inter-governmental organizational data as well as bridging data reflecting the relationships between providers and their outer contexts [59, 60]. We expect such information would be useful not only for research but have practical value as well.

There are other limitations relevant to this paper. We did not obtain one-third of the states’ SOR applications, so even though there were no important characteristics we could discern from states that did and did not provide their applications, there may still be some differences that we did not detect. Also, the modest sample size, the ordinal and dichotomous measures, along with the multiple univariate tests could have identified some spurious relationships. Indeed, a few relationships that were significant in univariate analysis were no longer significant in multiple regressions. Accounting somewhat more for the possibility of spurious findings, the machine learning analyses have built-in cross-validation procedures that minimize effects of overfitting.

From an implementation practice perspective, state addiction prevention and treatment system authorities need and want guidance on how to leverage change in policy, financing and regulations so that the best available programs are, and remain, accessible to their citizens across the entire system. Practical information for systems leaders about effective and efficient sustainment strategies is therefore sorely needed [61].

## Supporting information

S1 Appendix Quantitative analysis keywords opiods 2020.07.31. This file contains the keywords used in the text mining and the categories of each list of keywords.
(XLSX)

S1 Data (CSV)
